# Application of Whole Genome Sequencing to Understand Diversity and Presence of Genes Associated with Sanitizer Tolerance in *Listeria monocytogenes* from Produce Handling Sources

**DOI:** 10.3390/foods10102454

**Published:** 2021-10-14

**Authors:** Rebecca N. Bland, Jared D. Johnson, Joy G. Waite-Cusic, Alexandra J. Weisberg, Elizabeth R. Riutta, Jeff H. Chang, Jovana Kovacevic

**Affiliations:** 1 Food Innovation Center, Oregon State University, Portland, OR 97209, USA; rebecca.bland@oregonstate.edu; 2 Department of Food Science and Technology, Oregon State University, Corvallis, OR 97331, USA; jared.johnson@oregonstate.edu (J.D.J.); joy.waite-cusic@oregonstate.edu (J.G.W.-C.); 3 Department of Botany and Plant Pathology, Oregon State University, Corvallis, OR 97331, USA; alexandra.weisberg@oregonstate.edu (A.J.W.); riuttae@oregonstate.edu (E.R.R.); jeff.chang@oregonstate.edu (J.H.C.)

**Keywords:** *Listeria monocytogenes*, genomic diversity, sanitizer tolerance genes, produce handling environments

## Abstract

Recent listeriosis outbreaks linked to fresh produce suggest the need to better understand and mitigate *L. monocytogenes* contamination in packing and processing environments. Using whole genome sequencing (WGS) and phenotype screening assays for sanitizer tolerance, we characterized 48 *L.* *monocytogenes* isolates previously recovered from environmental samples in five produce handling facilities. Within the studied population there were 10 sequence types (STs) and 16 cgMLST types (CTs). Pairwise single nucleotide polymorphisms (SNPs) ranged from 0 to 3047 SNPs within a CT, revealing closely and distantly related isolates indicative of both sporadic and continuous contamination events within the facility. Within Facility 1, we identified a closely related cluster (0–2 SNPs) of isolates belonging to clonal complex 37 (CC37; CT9492), with isolates recovered during sampling events 1-year apart and in various locations inside and outside the facility. The accessory genome of these CC37 isolates varied from 94 to 210 genes. Notable genetic elements and mutations amongst the isolates included the *bcrABC* cassette (2/48), associated with QAC tolerance; mutations in the *actA* gene on the *Listeria* pathogenicity island (LIPI) 1 (20/48); presence of LIPI-3 (21/48) and LIPI-4 (23/48). This work highlights the potential use of WGS in tracing the pathogen within a facility and understanding properties of *L.* *monocytogenes* in produce settings.

## 1. Introduction

*Listeria monocytogenes* is a Gram-positive environmental pathogen, frequently associated with food processing environments [1,2,3]. Recent listeriosis outbreaks involving fresh produce have demonstrated serious foodborne illness risks for this food category [4,5,6,7,8]. Most notably, in 2011, a listeriosis outbreak linked to contaminated cantaloupe caused 147 illnesses and 33 deaths. Food and Drug Administration (FDA) investigations revealed that a lack of hygienic design of washing equipment was a likely cause for contamination and cross contamination of the implicated product [9]. With natural reservoirs in agricultural growing environments, the risk of *L. monocytogenes* in fresh produce packing houses and processing facilities is a challenge that the industry must address [10,11]. In many fresh produce facilities, growers are faced with a large volume of incoming raw product directly from the field and must handle it as quickly as possible to maintain optimum quality. Depending on the type of produce, this may include diverse operations such as shaking, sorting, trimming, washing, waxing, and often chilling. Some facilities will handle one or a few different crops from few sources, while others will handle many different crops from many different growers. Control of *L. monocytogenes* in fresh produce facilities is almost entirely reliant on cleaning and sanitation; however, many of these facilities do not have hygienically designed equipment nor a robust environmental monitoring program to mitigate and monitor their risks.

In facilities handling freshly harvested produce, there will likely be frequent detection of *Listeria*. This makes determining appropriate corrective actions difficult as positive samples may be due to transient *Listeria* coming in with product that will be effectively mitigated by cleaning and sanitation; or positive samples may be from a persistent *Listeria* population that would require investigation to find the harborage site [12].

Testing methods for environmental swabs used by the industry are often rapid screening methods [13,14,15,16] or standard culture methods (ISO 11290 [17] and BAM [18]) that report the results as positive or negative for either *Listeria* spp. or *L. monocytogenes.* Historically, the industry has been most interested in obtaining a result as quickly as possible; therefore, most results are presumptive and not confirmed. While this approach provides immediately actionable results, it fails to yield confirmed isolates that can be characterized to watch for meaningful trends to find persistent problems. Classification can range in cost and ease of data analysis from serotyping to whole genome sequencing (WGS). While WGS analysis can provide the highest level of discrimination, the food industry has been reluctant to embrace this approach due to fear of regulatory access and use of these data to support increased regulation or increased investigations. Less discriminatory and simpler characterization may have utility in monitoring for persistent *L. monocytogenes.* To effectively evaluate the suitability of each characterization option, these analytical tools need to be applied to a set of diverse isolates from different food commodity processing environments and geographic locations.

Our previous investigation of the prevalence of *Listeria* spp. and *L. monocytogenes* in produce processing facilities in the Pacific Northwest region of the United States [19] has yielded a set of isolates that supports this characterization. The objective of the current study was to evaluate the suitability of genotypic analyses (MLST, cgMLST, whole genome single nucleotide polymorphism [SNP], and pangenome analyses) to classify and differentiate *L. monocytogenes* isolates. Phenotypic stress response to a commercial quaternary ammonium compound (cQAC) was used in parallel to further understand and differentiate the highly related isolates in produce facility environments.

## 2. Materials and Methods

### 2.1. Whole Genome Sequencing and Data Assembly

*L. monocytogenes* isolates (n = 48) were previously isolated from environmental samples collected from produce handling and processing facilities in the Pacific Northwest region of the United States between May 2018 and April 2019. Isolate source and initial characterization are described by Jorgensen et al. [19,20]. Isolates were stored at −80 °C in tryptic soy broth (TSB; Neogen, Lansing, MI, USA) supplemented with 25% (wt/vol) glycerol. Prior to use, isolates were resuscitated by streaking onto tryptic soy agar (TSA; Neogen, Lansing, MI, USA) supplemented with 0.6% Yeast Extract (YE; Fisher, Hampton, NH, USA), followed by 24 h incubation at 37 °C.

Genomic DNA of the 48 isolates was extracted using the Qiagen DNeasy Blood and Tissue Kit (Qiagen; Hilden, Germany) according to manufacturer’s instructions for Gram-positive bacterial DNA. Quality was measured using the HS dsDNA assay kit (Fisher; Waltham, MA, USA) on the Qubit fluorometer (Fisher; Waltham, MA, USA). The quality of genetic material was determined using the nanodrop spectrophotometer (Fisher; Waltham, MA, USA). DNA libraries were prepared at Oregon State University Center for Quantitative Life Sciences (Corvallis, OR, USA) using the PlexWell kit (seqWell; Beverly, MA, USA) according to the manufacturer’s instructions. Libraries were sequenced using 2 × 150-bp paired-end sequencing on the Illumina HiSeq platform.

Raw reads were quality checked with FastQC (v 0.11.9; http://www.bioinformatics.babraham.ac.uk/projects/fastqc, accessed on 11 January 2021), followed by low-quality trimming using Trimmomatic (v 0.39; accessed on 5 May 2020 [21]). Trimmed reads were *de novo* assembled using SPAdes (v 3.14.1 [22]) optimized with Unicycler (v 0.4.8; accessed on 3 March 2021 [23]). Resulting assembly files were assessed for quality (Table S1) and annotated with Prokka (v 1.12; accessed on 5 May 2020 [24]).

### 2.2. Core Genome Multilocus Sequence Typing (cgMLST)

Core genome MLST (cgMLST) was performed in the BIGSdb-*Lm* platform, using a scheme consisting of 1748 conserved core genes [25]. After comparing isolates to cgMLST profiles already in the database, assemblies of all isolates were submitted to the *Listeria* Pasteur database to receive cgMLST type (CT) assignments.

### 2.3. Detection and Assessment of Antimicrobial Resistance, Stress Survival, and Pathogenicity Genes

All isolates were screened for presence or absence of antimicrobial resistance genes using NCBI BLASTN [26] with a minimum nucleotide identity and alignment length coverage of 80% (*tetR*, *tnpABC*, *qacH*, *qacC*, *emrE*, *emrC*, and *bcrABC*). Resistance determinants to the heavy metal cadmium were also assessed (*cadA1* and *cadA2*). Genomic investigation of virulence genes and genetic elements was carried out by screening for presence/absence and mutation within *Listeria* pathogenicity islands (LIPI-1, LIPI-3, LIPI-4) and internalin A (*inlA*). Similarly, presence/absence and mutations in stress survival islet SSI-1 was also evaluated in all isolates. Accession numbers for reference strains used for screening and alignments of all genes and genetic elements can be found in Supplementary Materials Table S2.

### 2.4. Whole Genome SNP and Pangenome Analyses

Single nucleotide polymorphisms (SNPs) were called as described by Weisberg, et al. [27] with slight modifications. Briefly, FastANI (v 1.1) was used to calculate pairwise average nucleotide identity (ANI) among isolates and confirm species-level grouping (i.e., *L. monocytogenes*; >95% ANI) [28]. Raw reads were mapped to a representative reference sequence within the group (WRLP472) using BWA mem (v 0.7.17; accessed on 20 August 2021 [29]). Alignments were annotated, sorted, and duplicate reads identified with Picard tools (v 2.0.1; accessed on 20 August 2021 [30]). Graphtyper (v 2.6.2; accessed on 20 August 2021) was run on the dataset with the default parameters [31]. SNPs were filtered using vcffilter in vcflib (v 1.0.0; accessed on 20 August 2021 [32]) with the options ‘-f “ABHet < 0.0 | ABHet > 0.33” -f “ABHom < 0.0 | ABHom > 0.97” -f “MaxAASR > 0.4” -f “MQ > 30”. SNP calls annotated as “FAIL” or “heterozygous” were filtered to “no-call”. Bcftools (v 1.3; accessed on 20 August 2021) was used to convert filtered SNP calls to fasta format [33]. The bitwise.dist function from the R package poppr (v 2.9.2; accessed on 20 August 2021) was used to construct pairwise SNP distance tables from the fasta alignments [34]. The R package poppr was used to construct and visualize a minimum spanning network.

Pangenome analysis was conducted with Panaroo (v 1.2.8; accessed on 20 August 2021 [35]) using Prokka annotations as input to obtain the core and accessory genome of the 48 *L. monocytogenes* isolates. Accessory genomes of each CT and isolates were determined using a custom python script (https://github.com/johnjare/panaroo_scripts/tree/main/population_genes; accessed on 25 July 2021).

### 2.5. Growth in the Presence of Quaternary Ammonium Compound (QAC) Sanitizer

Growth of 48 *L. monocytogenes* isolates in the presence or absence of a commercial quaternary ammonium compound (cQAC; Professional Lysol No Rinse Sanitizer; EPA registration 675-30; Reckitt Benckiser, Parsippany, NJ, USA) was evaluated. Prior to use, isolates were resuscitated by streaking onto TSA-YE (Neogen), followed by 24 h incubation at 37 °C. Stock solution of the cQAC sanitizer was prepared in accordance with the manufacturer’s recommended concentration (MRC; 200 ppm), filter sterilized and stored for up to one week at 4 °C. Cultures were added to TSB supplemented with yeast extract (TSB-YE) containing 1.56 ppm cQAC at an initial inoculum of approximately 7 log CFU/mL in a final volume of 10 mL. An aliquot (200 µL) of each culture in sanitizer was transferred to a sterile 96-well plate (VWR; Radnor, PA, USA), in duplicate. Plates were incubated at 30 °C in a SpectraMax plate reader (Molecular Devices) and OD_600_ was measured at 30 min interval for 24 h with shaking (5 s) prior to each measurement. The OD_600_ data were fitted to growth curves to obtain the lag phase duration (LPD), maximum growth rate (MGR), and maximum density (OD) using the DMFit 3.0 Excel add-in program (ComBase; Computational Microbiology Research Group, Institute of Food Research, Colney, Norwich, UK), based on the models of Baranyi and Roberts [36]. All statistical analyses were performed in JMP (Version 16.0.0, SAS Institute Inc., Cary, NC, USA). One-way ANOVA was performed on lag phase and growth rate data to evaluate differences between isolates and cgMLST types when exposed to sub-lethal concentrations of cQAC. Tukey-Kramer HSD was used as a post-hoc test to determine significant differences between change in lag phase and growth rate by strain and cgMLST types. Students *t*-test was used to evaluate differences between growth parameters of control (without sanitizer) and 1.56 ppm of the sanitizer compound.

## 3. Results

### 3.1. General Genome Characteristics

**Figure 1:**
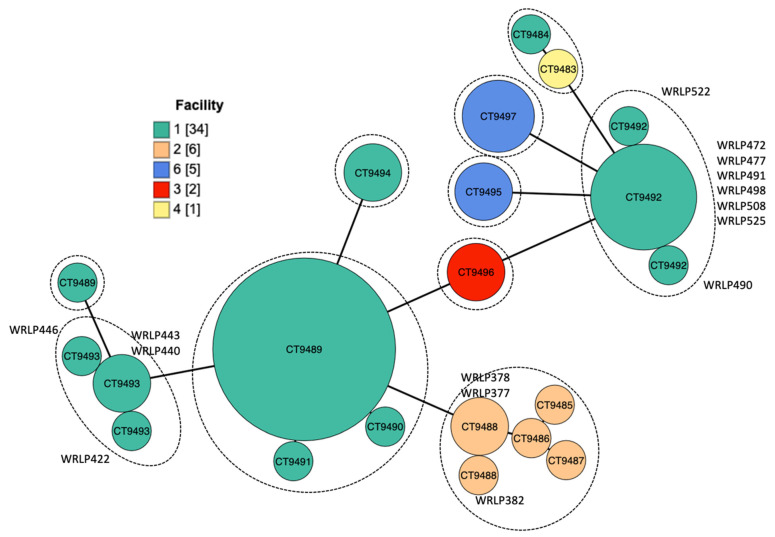
Minimum spanning tree of core genome MLST (cgMLST) profiles. Unique profiles are represented by nodes, the size of each node reflects the number of isolates that share an identical cgMLST profile. CT represented by different nodes have up to seven allelic differences. Colors are representative of the facility number from which the isolate was found. The length of the branch between nodes is proportional to the number of allelic differences between profiles. The dashed circles groupings of the nodes represent the sublineages which have up to 150 allelic differences.

*L. monocytogenes* isolates (n = 48) were grouped into 10 MLST profiles (ST) and were associated with 10 clonal complexes (CCs) (Table 1). Lineage I isolates included six CCs, while four CCs were observed among lineage II isolates. Core genome MLST (cgMLST) further delineated the 48 isolates into 16 cgMLST types (CT; Figure 1). The BIGSdb-*Lm* typing scheme defines cgMLST types as groups of profiles that differ by up to seven allelic types out of the 1748 loci, and sub-lineages (SL) differing by up to 150. The SL assignments were all in agreement with the classic MLST typing scheme determined and previously published that considers just seven housekeeping genes; therefore, ST and cgMLST types will be used to describe isolates [20,37].

*Listeria* pathogenicity islands LIPI-1, LIPI-3, and LIPI-4 were found amongst the isolates in this study (Table 1). LIPI-1, which harbors six of the virulence genes associated with listeriosis infection (*prfA*, *plcA*, *hly*, *mpl*, *actA*, and *plcB*), was present in all isolates. In all isolates belonging to CC345/ST2165 and CC4/ST219, the *actA* gene had a 35 amino acid in-frame deletion at nucleotide position 793 [38]. Previously reported deletions in the *prfA* gene leading to attenuated virulence were not observed [39]. LIPI-3 were absent from all lineage II (serotype 1/2a or 3a) isolates, as well as CC2/ST2, CC388/ST388, and CC345/ST2165 within lineage I. LIPI-3 (*llsG*, *llsH*, *llsX*, *llsB*, *llsY*, *llsD*, *llsP*, *llsA*), which codes for hemolytic and cytotoxic factors important in gastrointestinal colonization [40], was present in 21/48 isolates. Isolates belonging to CC4, CC6, and CC668 from Facility 1 harbored LIPI-3. LIPI-4 was only present in a subset of lineage I isolates (ST219, ST388, and ST688) within CC4, CC388, and CC688. Isolates harboring both LIPI-3 and 4 belonged to CC4/ST219 and CC688/ST688.

### 3.2. SNP Analyses

**Figure 2:**
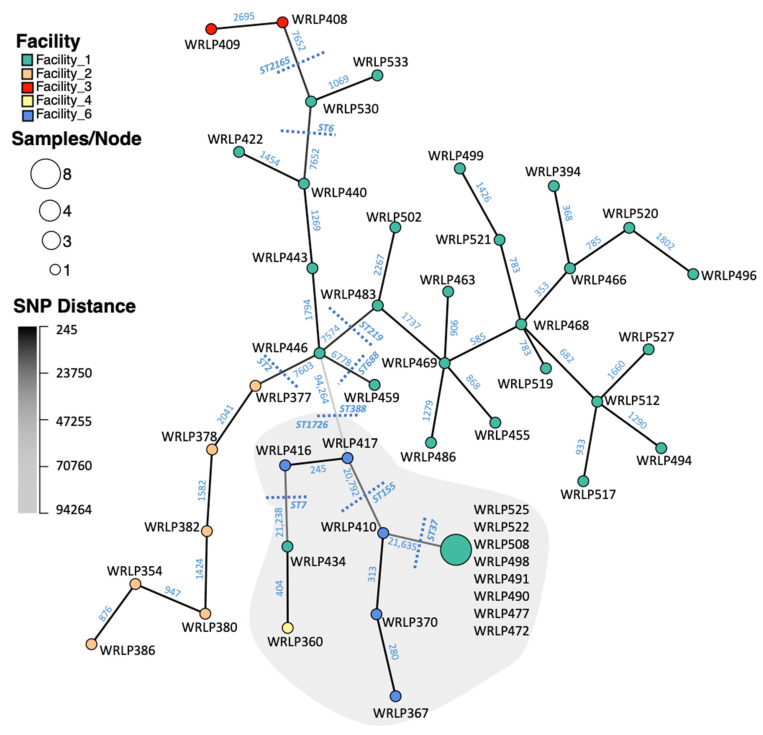
Minimum spanning network (MSN) of whole genome SNPs identified amongst 48 *Listeria monocytogenes* genomes. Nodes represent genotypes of isolates with <3 pairwise SNP differences and node size is proportional to the number of isolates of that genotype. Node color indicates the produce facility where each strain was sourced. Branch color and bales indicate the number of pairwise SNPs between genotypes (i.e., darker colors indicate fewer differences). Dashed lines illustrate where ST groupings fall in relation to the minimum spanning network. Grey background shows isolates belonging to lineage II (serotype 1/2a or 3a) whereas a white background shows isolates belonging to lineage I (serotype 4b, 4d, or 4e).

Single nucleotide polymorphisms (SNPs) of whole-genome sequences (WGS) were used to characterize the relationship amongst the isolates within and between the produce processing facilities (Figure S1). The minimum spanning network (MSN) in Figure 2 illustrates the relative similarity between isolates of the largest ANI species group, with nodes (representing genotypes) connected by a branch to the node that has the fewest SNP differences (i.e., most similar). Isolates of the two serotypes (1/2a and 4b) were seen to cluster together based on having relatively few SNP differences. The branch connecting WRLP446 and WRLP417 is light grey, signifying a large number of SNP differences (94,264) at the breakpoint between lineage I and II in the tree. Isolates belonging to the same ST and CC are clustered within the tree.

The genomes of the 48 *L. monocytogenes* isolates differed by as few as 0 SNPs and up to 120,331 SNPs (WRLP386, lineage I; and WRLP508, lineage II). Within each CT, SNP differences ranged from 0 to 3047 SNPs. Within each CT, pairwise SNP differences between isolates ranged from 0 to 2 (CT9492); 280 to 415 (CT9497); 245 (CT9495); 2069 to 2196 (CT9488); 1069 (CT9494); 1269 to 1887 (CT9493); to 353 to 3047 (CT9489). Isolates within a facility differed by 0 to 118,686 SNPs (Facility 1); 876 to 2818 SNPs (Facility 2); 2695 SNPs (Facility 3; only two isolates); and 245 to 22,863 SNPs (Facility 6). The only ST found within multiple facilities was ST7, isolated from both Facility 1 (WRLP434) and Facility 4 (WRLP360). These isolates had a total of 404 pairwise SNP differences and belonged to two different CTs based on core genome allelic differences.

### 3.3. Highly Related CC37 Cluster in Facility 1

**Figure 3:**
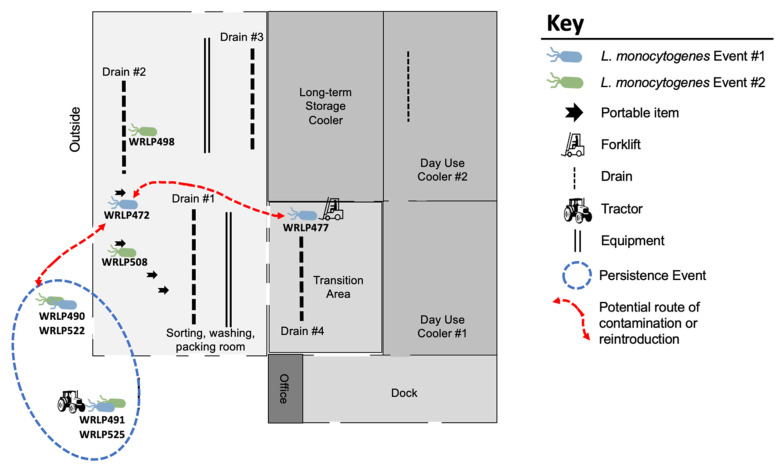
Sampling locations of isolates belonging to CC37 found throughout the facility, with 0–2 SNP differences over two different sampling events. Blue circle depicts suspected source of persistent *L. monocytogenes* and red arrows show a potential route of contamination into the facility.

Within Facility 1, SNP analysis revealed high similarity amongst CC37 (ST37), with a maximum of two SNP differences seen between any two isolates in the ST (Figure 2). Isolates belonging to ST37 originated from samples taken during two different sampling events roughly one-year apart (Figure 3). Within this CC, identical isolates (0 pairwise SNPs; WRLP491, WRLP525) were found on a tractor tire immediately outside of the facility collected during two separate sampling events. Of the samples taken from across the facility on mobile elements, inside a drain, and on a forklift tire, 3/4 (WRLP508, WRLP472, and WRLP477) isolates had no pairwise SNPs amongst themselves and compared to the isolates recovered outside the facility on the tractor tire (Figure 3). The remaining three isolates, found immediately outside of the facility and in a drain inside the facility, had from one to two pairwise SNP differences between other isolates within the CC (WRLP490, WRLP498, and WRLP522).

### 3.4. Accessory Genome Analysis

The 48 isolates, recovered from five different facilities, shared a core genome of 2391 genes and a pangenome of 9861 genes. The accessory genome, reflecting genes unique to a single isolate or group, was evaluated for each ST/CC, as well as for individual isolates within their respective ST/CC (Table 2). The number of genes in the accessory genome of a CC ranged from 3 to 103 genes. When isolates belonging to the same CC were compared, the number of their accessory genes ranged from 1 to 683 genes. Isolates within CC2 (WRLP354, WRLP377, WRLP378, WRLP380, WRLP382, and WRLP386) had a nearly identical accessory genome with only 1 to 12 accessory gene differences.

### 3.5. Phenotypic Analyses and Presence of Genetic Elements Assocaited with QAC Tolerance

**Figure 4:**
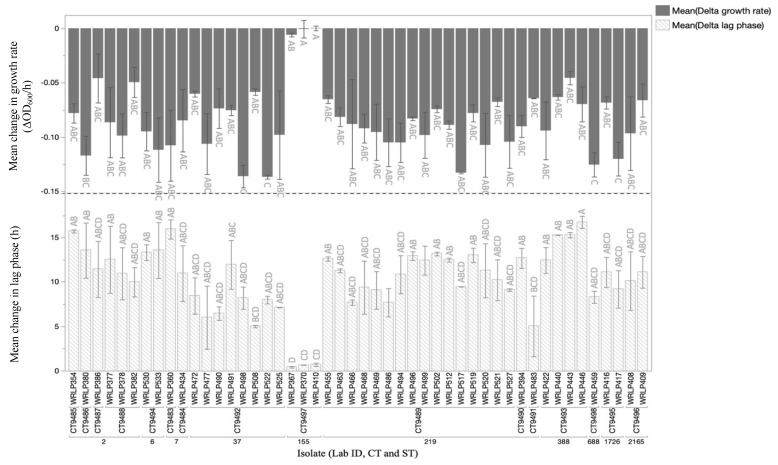
Average change in growth rate (ΔOD_600_/h; dark grey bars) and lag phase duration (h; striped bars) in the presence of 1.56 ppm commercial quaternary ammonium compound-based sanitizer compared to the growth rate and lag phase duration in the absence of the sanitizer. Isolates are clustered by multilocus sequence types (ST) and core genome MLST types (CT). Error bars represent standard error (n = 2). Isolates with different letter above (change in lag phase) or below (change in growth rate) their respective bar are significantly different based on the means comparisons of all pairs using ANOVA with Tukey-Kramer HSD at *p* < 0.05.

When isolates were grown in the presence of cQAC at 1.56 ppm, the lag phase duration and growth rate were significantly impacted for 45/48 *L. monocytogenes* strains (Figure 4). These 45 isolates all showed significantly longer lag phase durations, and significantly slower growth rates compared to growth in the absence of the sanitizer (*p* < 0.05; student’s *t*-test). The growth of three strains, all belonging to CC155/CT9497, was only minimally influenced by the presence of cQAC, with no significant reduction in growth rate for any of the isolates within this CC (WRLP367, WRLP370, WRLP410; *p* < 0.05 student’s t-test). Genetic determinants of QAC tolerance that may explain the phenotype observed, such as specific efflux pumps (i.e., *bcrABC)*, were only found in 2/48 isolates (WRLP370, WRLP410). The two isolates in which the *bcrABC* cassette was found belonged to the same CC (CC155) and CT (CT9489) and were both recovered from Facility 6. Notably, the third isolate demonstrating QAC tolerance (WRLP367) did not possess the *bcrABC* cassette despite being a member of the same CC/CT and also being isolated from the same facility. Stress survival islet 1 (SSI-1: *lmo0444*, *lmo0445*, *lmo0446*, *lmo0447*, *lmo0448*) was found in 4/48 isolates. Of these four isolates, three belonged to CC155 and exhibited phenotypic tolerance towards cQAC (no significant change in growth rate). The SSI-1 harbored by each of the isolates within CC155 possessed an insertion of 4 bp at nucleotide position 2571 within *lmo0444* resulting in a premature stop codon (PMSC). No insertion was found within the only other isolates harboring SSL-1 (WRLP360, CC7). Isolates within CC155 had 61 unique genes compared to other isolates within lineage II and 39 unique genes when compared to the entire isolate set. Each isolate within the ST had between 103 and 536 genes unique to the other isolates belonging to CC155. Gene predictions of the genes within the accessory genome of CC155 revealed that there are several transcriptional regulators, as well as genes predicted to code for membrane components (Table S3).

The high similarity amongst isolates in ST37/CT9492 (WRLP472, WRLP477, WRLP490, WRLP491, WRLP498, WRLP508, WRLP522, and WRLP525) from Facility 1 suggested its potential persistence; therefore, the evaluation of sanitizer tolerance was of particular interest. None of these strains were particularly tolerant to cQAC (Figure 4).

The majority (68%) of *L. monocytogenes* isolates in this study carried genes associated with resistance towards the heavy metal cadmium (*cadA1* and *cadA2*) (Table 1). One or both cadmium resistance determinants were found in all lineage I isolates, and were absent from all lineage II isolates.

## 4. Discussion

The risk of *L. monocytogenes* in fresh produce remains a concern due to its natural ecology in agricultural environments and the lack of a kill step in produce production. While public health and regulatory agencies have embraced WGS for outbreak investigations, the use of this technology to determine the biodiversity and assist in the understanding environmental sampling data in produce environments is not frequently employed by the industry. The *L. monocytogenes* isolates used in this study were obtained through environmental sampling of produce processing and handling facilities in the Pacific Northwest region of the United States [19]. While the limited number of isolates used in this study makes it difficult to draw conclusions about the region or industry as a whole, the data provide insight into the diversity, relatedness, and virulence potential of *L. monocytogenes* within produce operations and highlight the levels of discrimination attainable through the use of WGS.

Initial grouping of the isolates using MLST and cgMLST resulted in 10 ST/CCs and 16 CTs, respectively. Three most frequently found ST/CCs included CC4/ST219, CC37/ST37, CC2/ST2. These CCs were also common among French isolates recovered from both food and clinical sources in the period from 2005 to 2013 [41]. The size of the core genome within our isolate population (2391 genes) is in line with the size of other reported core genomes for *L. monocytogenes* (2322 to 2562 genes) [39,42,43,44,45,46]. The accessory genome in the present study, which generally encompasses a larger pool of genes compared to the core genome, included 7470 genes. A recent study that looked at the pangenome of *L. monocytogenes* from soil samples across the United States suggested that pangenome openness is the consequence of adaptive evolutionary processes that enable bacteria to survive in variable environments [45]. This study found that the pangenome and especially accessory genes of *L. monocytogenes* evolve at a faster rate than the core genome is seen to diversify [45]. The authors suggest that because of this difference in diversification rate, the accessory genome can be used as a further measure of diversity within a set of isolates [45]. Within our isolate set, a number of CC37 isolates possessing 0–2 pairwise SNP differences appeared to be identical or highly similar (Figure 2). However, the accessory genome for each isolate within this CC differed by 94–210 genes (Table 2). The variance in cQAC tolerance as well as the differences in antibiotic resistance profiles (Table 1) also suggest that these strains behave differently. While these differences are not explained by SNPs, the accessory genome of each isolate within CC37 suggests that these genes may be giving rise to the phenotypic differences observed. Further investigation is necessary to understand the variation in predicted gene function of these isolates and to infer what advantage they may be contributing to survival. The isolation of these highly similar CC37 isolates immediately outside the facility from multiple sampling events and dissemination across the facility (Figure 3), suggests continuous contamination into the processing environment and routes of dissemination throughout the facility. Isolation of related isolates (WRLP491 and WRLP525) from the tractor tire over two different sampling events is not unexpected as any *Listeria* contamination within the facility is most likely coming from the growing environment, which for these facilities was relatively local. All sampling events of isolates included in this study were conducted during production. Therefore, the presence of closely related isolates, such as in CC37 could be due to recurring contamination from a field, recurring introduction into the facility from an outside harborage site, or a potential persistence event within the facility.

In the present study, both *cadA1* and *cadA2*, genes associated with cadmium resistance [47], were found in all lineage I isolates and 68% of total isolates. Heavy metals such as cadmium can be used in various pesticide products (including sanitizers) and therefore are commonly found in soils, and agricultural environments. Bacterial populations within these environments, such as *L. monocytogenes*, often possess genetic elements to combat this stressor [47,48]. These genes are commonly found on mobile genetic elements, such as plasmids (e.g., pLM80) or transposons (e.g., Tn*5422*), and their proximity in coding regions to QAC tolerance determinants (e.g., *bcrABC*) has led to speculation that the two may be associated. However, there are no data to suggest that the presence of one (e.g., QAC tolerance determinant) influences the other (i.e., cadmium tolerance determinant) or vice versa [49,50]. In the present study all ST2 and ST2165 isolates possessed both *cadA1* and *cadA2*, whereas isolates belonging to ST219, ST388, and ST6 were found to only have *cadA2* (31/48). The presence of *cad* did not seem to impact QAC tolerance. In evaluating isolates from food products, clinical cases, and environmental samples for QAC and heavy metal resistance genes, Gelbicova, et al. [48] found that cadmium resistance determinants were more frequently found across the samples compared to QAC tolerance genes (27.8% vs. 7%). While the prevalence rate of cadmium resistance and QAC tolerance genes was similar in the present sample set, the combination of both gene types was not found in any one isolate (i.e., isolates with *bcrABC* did not have either *cadA1* or *cadA2*). The presence of these resistance determinants, especially those carried on mobile elements, is of particular concern due to possible horizontal gene transfer in the population and between species [50].

To better understand the behavior of our isolates in the presence of cQAC we exposed them to a sublethal concentration of cQAC. Three CC155 isolates had no significant decrease in growth rate and two isolates had no significant increase in lag phase duration (WRLP367, WRLP410), suggesting increased tolerance to cQAC (Figure 4). This phenotypic tolerance is in part explained by the presence of the *bcrABC* cassette found in WRLP370 and WRLP410 and is in line with previous reports of this element conferring increased tolerance to benzalkonium chloride and commercial QAC products [49,51,52,53,54,55]. Initially found to be harbored in an outbreak strain from the 1998-1999 listeriosis outbreak in hot dogs, Elhanafi, et al. [51] and Dutta, et al. [49] described a three-gene cassette, with *bcrA* and *bcrB* coding for small multidrug resistance (SMR) transporters and *bcrC* as a transcriptional regulator. Elhanafi, et al. [51] showed that the transcription levels of *bcrABC* are higher in cultures exposed to subinhibitory concentrations of QAC than in cultures grown without QAC. Dutta, et al. [49] noted a strong association of benzalkonium chloride tolerance in isolates that harbor this element (70/71 strains). The tolerance of both WRLP370 and WRLP410 to cQAC can in part be explained by the presence of the *bcrABC* cassette. However, WRLP367 behaved similarly to the other two isolates within CC155, but it lacked any known genetic elements associated with QAC tolerance. Other studies have also reported QAC tolerance in the absence of common genetic elements associated with QAC tolerance, such as *bcrABC*, *qacH*, *qacC*, or *emrE* [56,57]. In some of these strains, general efflux pumps found across the species, such as *mdrL* and *lde*, and modifications to reduce the cell membrane permeability have been proposed to be contributing factors to QAC tolerance, thought they are not consistently reported [58,59,60,61].

The presence of SSI-1, associated with increased tolerance to low pH and high salt stress, has also been described in isolates with phenotypic tolerance to QAC [62]. Particularly, the occurrence of this islet within CC155 has been previously reported [3,39,56]. The high prevalence of CC155 among food, food processing and clinical cases, indicates its importance and warrants further exploration [39,41]. Notably, we observed the 4 bp deletion within *lmo0444* (SSI-1), resulting in a PMSC in three isolates that exhibited phenotypic tolerance to cQAC (WRLP367, WRLP370, WRLP410), whereas the only other isolate that harbored the SSI-1 (WRLP360; CC7/ST7) encoded the full-length gene. Ryan, et al. [62] speculated through in silico analysis that *lmo0444* encodes a hypothetical protein with a reovirus attachment protein domain and possesses homology with a phage infection protein. Further analysis is needed to elucidate the effect of this deletion on the function of the gene and impact on the islet as a whole on resulting stress tolerance. Additionally, Keeney, et al. [63] reported that the presence of SSI-1 was strongly correlated with biofilm forming ability as shown by increased adherence in adhesion assays. Other research has suggested that genetic determinants associated with QAC tolerance confer tolerance, rather than resistance, to sanitizers used within food processing environments [64]. However, the impact of biofilm formation on persistence within a facility and increased tolerance towards QAC has been described [65]. It is likely that the combination of a set of genes conferring increased biofilm formation in combination with a sanitizer stress efflux system may confer increased overall survival within a facility.

When considering the virulence potential of *L. monocytogenes*, intact *inlA* and pathogenicity islands have been accepted as common virulence markers [66,67,68,69]. No PMSCs in *inlA* were detected among any of the 48 tested isolates suggesting fully functional genes. Previous literature has reported a relatively high rate of mutations leading to PMSC across lineage II isolates (~30%), whereas linage I is more likely to harbor a full-length gene [66,67,68]. The disproportional representation of lineage I isolates within the present data set may be in part responsible for the lack of mutations seen here. Nightingale, et al. [68] illustrated that invasion efficacies of strains with an *inlA* PMSC were reduced (from −3.6 to −4.24) compared to those of full-length genes (from 0.52 to 0.89). While no PMSCs were detected in the isolates studied here, a 9 bp deletion within *inlA* was found in WRLP530 and WRLP533 of CC6/ST6. This mutation was initially reported by Kovacevic, et al. [70] and has since been reported by Kanki, et al. [71], Smith, et al. [72], and Raschle, et al. [42]. Raschle, et al. [42] found 2/25 isolates from surface water in Switzerland possessing this 9 bp deletion, both of which also belonged to CC6 and were the only two CC6 isolates within the sample set. Similarly, Smith, et al. [72] found this mutation in the only isolate belonging to CC6 within a sample set from produce and produce processing environments. Kovacevic, et al. [70] and Kanki, et al. [71] both found that isolates with this deletion had equivalent or increased invasion efficacy to isolates encoding the full-length *inlA*. While the mutations seen within this gene may not be leading to a reduction in virulence, Keeney, et al. [63] found that this mutation was correlated with a significant decrease in the ability to form biofilms. The combination of increased ability to survive within a food processing facility and increased ability to cause disease in the event of consumption of contaminated food product is of particular concern.

Further evidence of virulence potential was assessed by screening for four major pathogenicity islands (LIPI1-4), associated with genes that increase the ability of *L. monocytogenes* to cause infection in a human host. *Listeria* pathogenicity island 1 (LIPI-1) is widely conserved across the species, harboring genes largely responsible for phagosome escape from host cells and cell-to-cell spread. Specifically, the *actA* gene within LIPI-1 is responsible for the formation of an actin tail, assisting in cell-to-cell spread [73,74]. The 35 amino acid *actA* deletion seen across the majority of lineage I isolates in our study has previously been described in other *L. monocytogenes* isolates [38,75,76]. This deletion was first identified in a dairy isolate by Jiang, et al. [38]. They reported similar invasion efficacy for both control and deletion strains in HeLa cell models, but a defect in cell-to-cell spread was observed in a plaque-forming assay (L929 cell line) [38]. A study by Holch, et al. [75] used a similar assay to evaluate cell-to-cell spread in isolates possessing this deletion from food, clinical and environmental sources. Conversely, they did not see that the deletion influenced the plaque formation associated with cell-to-cell spread. The deletion allele only possesses three of four protein-rich repeats within the proline rich region of the gene. Smith, et al. [73] detailed the effect of deletions of these repeats as being largely responsible for motility [73]. Further analysis is needed to determine if similar virulence potential is observed in the strains within the present study. The occurrence of this deletion within isolates otherwise associated with increased virulence (lineage I, CC4, LIPI-3, and LIPI-4), highlight the genomic complexities found within produce environments.

Other pathogenicity islands, LIPI-3 and LIPI-4 were found in 44% (21/48) and 48% (23/48) of our isolates, respectively. Both islands were seen across all CC4 and CC688 isolates (19/48). The prevalence of these islands in lineage I CTs and more specifically LIPI-4 in CC4 is in line with what is reported in the literature [42,69]. A recent study by Maury, et al. [69] found that the six genes on LIPI-4 are associated with enhanced invasion, central nervous system and maternal-neonatal listeriosis and are strongly associated with CC4. While most virulence genes are well conserved across *L. monocytogenes*, the presence of pathogenicity islands LIPI-3 and LIPI-4, both of which are strongly associated with lineage I isolates, are not always present across the species [40,41,77]. Their prevalence within the present set of data suggests their increased virulence potential.

## 5. Conclusions

By evaluating a set of *L. monocytogenes* previously isolated from produce processing facilities we were able to examine the utility of various genomic analyses on their ability to differentiate isolates within produce environments in aiding our understanding of contamination events. Within environments that contain closely related strains, a deeper level of analysis, likely beyond MLST and even cgMLST, may be needed to differentiate potential persistent or recurring strains from those that are transient. Whole genome SNP analysis and pangenome analysis resulted in similar classifications that could separate “nearly identical” (i.e., likely persistent) and “clearly different” (i.e., likely transient) strains, which is a necessary level of interpretation for optimizing the use of environmental monitoring programs. The identification of a “nearly identical” (likely persistent) population in one facility identified a potential route of contamination and suggested the presence of a harborage site in an outdoor high traffic area that seems to be a source of indoor contamination on more than one processing day.

While SNP analysis was useful in discriminating between isolates, and it is generally considered as an important tool in epidemiological traceback and outbreak investigations, it did not fully provide insight into isolate properties and differences, such as those seen through phenotypic responses and the accessory genome. Our data show that fully exploiting WGS data and considering virulence profiles, SNPs, and accessory genomes can lead to a greater understanding of closely related populations that may be missed otherwise. By pairing genotypic data with some basic phenotypic analysis in the form of susceptibility profiling to antimicrobials is suggestive of genomic differences and helps guide and confirm risk management decisions.

## Figures and Tables

**Table 1:** Distribution of antimicrobial resistance genes and virulence factors across the isolates.

Isolate ID	Lineage ^a^	ST	CC	cgMLST	Fac.#	*bcrABC*	SSI-1 ^b^	*cadA* Type	LIPI1 ^c^	LIPI3	LIPI4	*inlA* ^d^	AMR Profile ^e^
WRLP360	2	7	7	CT9483	4	-	+	*-*	+	-	-	+	CLI
WRLP434	2	7	7	CT9484	1	-	-	*-*	+	-	-	+	CLI
WRLP472	2	37	37	CT9492	1	-	-	*-*	+	-	-	+	PEN
WRLP477	2	37	37	CT9492	1	-	-	*-*	+	-	-	+	PEN
WRLP490	2	37	37	CT9492	1	-	-	*-*	+	-	-	+	CLI, PEN
WRLP491	2	37	37	CT9492	1	-	-	*-*	+	-	-	+	CLI, PEN
WRLP498	2	37	37	CT9492	1	-	-	*-*	+	-	-	+	
WRLP508	2	37	37	CT9492	1	-	-	*-*	+	-	-	+	
WRLP522	2	37	37	CT9492	1	-	-	*-*	+	-	-	+	
WRLP525	2	37	37	CT9492	1	-	-	*-*	+	-	-	+	
WRLP367	2	155	155	CT9497	6	-	+Δ	*-*	+	-	-	+	CLI
WRLP370	2	155	155	CT9497	6	+	+Δ	*-*	+	-	-	+	CLI
WRLP410	2	155	155	CT9497	6	+	+Δ	*-*	+	-	-	+	CIP, CLI
WRLP417	2	1726	452	CT9495	6	-	-	*-*	+	-	-	+	CHL, CIP, NOV
WRLP416	2	1726	452	CT9495	6	-	-	*-*	+	-	-	+	CHL, CIP, PEN
WRLP354	1	2	2	CT9485	2	-	-	*A*1, *A*2	+	-	-	+	CLI
WRLP380	1	2	2	CT9486	2	-	-	*A*1, *A*2	+	-	-	+	AMP, CIP, PEN
WRLP386	1	2	2	CT9487	2	-	-	*A*1, *A*2	+	-	-	+	CLI, PEN
WRLP377	1	2	2	CT9488	2	-	-	*A*1, *A*2	+	-	-	+	
WRLP378	1	2	2	CT9488	2	-	-	*A*1, *A*2	+	-	-	+	CLI
WRLP382	1	2	2	CT9488	2	-	-	*A*1, *A*2	+	-	-	+	CIP, CLI
WRLP530	1	6	6	CT9494	1	-	-	*A*2	+	+	-	Δ	CLI
WRLP533	1	6	6	CT9494	1	-	-	*A*2	+	+	-	Δ	CLI
WRLP499	1	219	4	CT9489	1	-	-	*A*2	+Δ	+	+	+	
WRLP521	1	219	4	CT9489	1	-	-	*A*2	+Δ	+	+	+	CLI
WRLP455	1	219	4	CT9489	1	-	-	*A*2	+Δ	+	+	+	CLI, PEN
WRLP463	1	219	4	CT9489	1	-	-	*A*2	+Δ	+	+	+	CLI, PEN
WRLP466	1	219	4	CT9489	1	-	-	*A*2	+Δ	+	+	+	CLI, PEN
WRLP468	1	219	4	CT9489	1	-	-	*A*2	+Δ	+	+	+	CLI, PEN, NOV
WRLP469	1	219	4	CT9489	1	-	-	*A*2	+Δ	+	+	+	CLI, PEN, NOV
WRLP486	1	219	4	CT9489	1	-	-	*A*2	+Δ	+	+	+	CLI, PEN
WRLP494	1	219	4	CT9489	1	-	-	*A*2	+Δ	+	+	+	CLI, PEN
WRLP496	1	219	4	CT9489	1	-	-	*A*2	+Δ	+	+	+	
WRLP502	1	219	4	CT9489	1	-	-	*A*2	+Δ	+	+	+	
WRLP512	1	219	4	CT9489	1	-	-	*A*2	+Δ	+	+	+	
WRLP517	1	219	4	CT9489	1	-	-	*A*2	+Δ	+	+	+	CLI
WRLP519	1	219	4	CT9489	1	-	-	*A*2	+Δ	+	+	+	CLI
WRLP520	1	219	4	CT9489	1	-	-	*A*2	+Δ	+	+	+	CLI, PEN
WRLP527	1	219	4	CT9489	1	-	-	*A*2	+Δ	+	+	+	CLI
WRLP394	1	219	4	CT9490	1	-	-	*A*2	+Δ	+	+	+	CLI
WRLP483	1	219	4	CT9491	1	-	-	*A*2	+Δ	+	+	+	PEN, NOV
WRLP422	1	388	388	CT9493	1	-	-	*A*2	+	-	+	+	CLI
WRLP440	1	388	388	CT9493	1	-	-	*A*2	+	-	+	+	CLI
WRLP443	1	388	388	CT9493	1	-	-	*A*2	+	-	+	+	AMP, CLI
WRLP446	1	388	388	CT9493	1	-	-	*A*2	+	-	+	+	CLI
WRLP459	1	688	688	CT9498	1	-		*A*2	+	+	+	+	CLI, NOV
WRLP408	1	2165	345	CT9496	3	-	-	*A*1, *A*2	+Δ	-	-	+	CIP, CLI, NOV
WRLP409	1	2165	345	CT9496	3	-	-	*A*1, *A*2	+Δ	-	-	+	CIP, CLI

^a^ Serotypes of each lineage include lineage I: 4b, 4d, or 4e and lineage II: 1/2a or 3a. ^b^ + presence of SSI-1; Δ internal 4 bp insertion in *lmo0444* resulting in a premature stop codon (PMSC). ^c^ + presence of LIPI-1; Δ 35 amino acid in frame deletion on *actA*. ^d^ + full length *inlA*, Δ internal deletion of 9 bp at nucleotide position 2212. ^e^ Resistance profiles previously determined by Jorgensen et al. (2021): AMP = ampicillin, CHL = chloramphenicol, CIP = ciprofloxacin, CLI = clindamycin, PEN = penicillin, NOV = novobiocin. All isolates were resistant to cefoxitin and nalidixic acid, blank rows indicate resistance to only these antibiotics.

**Table 2:** Evaluation of accessory genomes between *L. monocytogenes* isolates (n = 48). The number of unique genes indicates the number of genes that were present in all genomes within the grouping (strain, ST, or lineage), but absent outside the respective grouping.

Isolate Grouping (Lineage/Serogroup; Sequence Type; Strain ID)	No. Unique Genes ^a^
**Lineage I/Serogroup 4b, 4d, 4e**	**11**
**ST7**	**20**
WRLP360	62
WRLP434	16
**ST37**	**49**
WRLP472	118
WRLP477	143
WRLP490	108
WRLP491	141
WRLP498	96
WRLP508	94
WRLP522	139
WRLP525	210
**ST155**	**39**
WRLP367	103
WRLP370	239
WRLP410	536
**ST1726**	**47**
WRLP416	406
WRLP417	46
**Lineage II/Serogroup 1/2a or 3a**	**104**
**ST2**	**36**
WRLP354	12
WRLP377	1
WRLP378	7
WRLP380	1
WRLP382	4
WRLP386	13
**ST6**	**21**
WRLP530	509
WRLP533	308
**ST219**	**3**
WRLP394	22
WRLP455	41
WRLP463	72
WRLP466	26
WRLP468	30
WRLP469	26
WRLP483	110
WRLP486	75
WRLP494	101
WRLP496	70
WRLP499	64
WRLP502	1
WRLP512	52
WRLP517	67
WRLP519	83
WRLP520	56
WRLP521	46
WRLP527	51
**ST388**	**9**
WRLP422	212
WRLP440	129
WRLP443	173
WRLP446	683
**ST688**	**19**
WRLP459	nd ^b^
**ST2165**	**103**
WRLP408	157
WRLP409	530

^a^ Number of genes unique to lineage and serogroup, sequence type or individual strains within each level of grouping. ^b^ nd, no data as there was only one isolate from this ST and comparison between isolates of the same ST was not possible.

## Data Availability

Not applicable.

## Supplementary Materials

The following are available online at https://www.mdpi.com/article/10.3390/foods10102454/s1, Table S1: Assembly statistics resulting from SPAdes *de novo* assemblies, Table S2: NCBI Accession numbers used to screen for antimicrobials tolerance determinants and virulence genes, Table S3: GoTerm analysis of select CC155 accessory genes annotated as DNA binding, transcriptional regulator or membrane component, Figure S1: Pairwise SNP matrix.
