# Supplementary material for: Application of Whole Genome Sequencing to Understand Diversity and Presence of Genes Associated with Sanitizer Tolerance in Listeria monocytogenes from Produce Handling Sources
Source: Foods. 2021 Oct 14;10(10):2454. doi: 10.3390/foods10102454 (PMC8536156; doi:10.3390/foods10102454)
Supplement: Supplementary file 1 [file foods-10-02454-s001.zip › foods-1402341-supplementary.pdf]

foods MDPI

Supplementary Materials

Table S1. Assembly statistics resulting from SPAdes *de novo* assemblies.

Strain ID	Species	Facility	Contigs No.	Length (bp)	N50
WRLP354	*L. monocytogenes*	2	38	3066783	296097
WRLP360	*L. monocytogenes*	4	24	2881261	1457222
WRLP367	*L. monocytogenes*	6	19	2889889	684358
WRLP370	*L. monocytogenes*	6	25	2979345	541396
WRLP377	*L. monocytogenes*	2	38	3038585	330851
WRLP378	*L. monocytogenes*	2	44	3037562	330851
WRLP380	*L. monocytogenes*	2	54	3103663	225002
WRLP382	*L. monocytogenes*	2	43	3037571	330851
WRLP386	*L. monocytogenes*	2	58	3103288	272216
WRLP394	*L. monocytogenes*	1	38	2927271	476657
WRLP408	*L. monocytogenes*	3	48	3082589	399511
WRLP409	*L. monocytogenes*	3	48	3082292	399511
WRLP410	*L. monocytogenes*	6	33	2978884	524000
WRLP417	*L. monocytogenes*	6	51	2926135	541208
WRLP416	*L. monocytogenes*	6	53	2925733	541208
WRLP422	*L. monocytogenes*	1	30	2892522	498434
WRLP434	*L. monocytogenes*	1	17	2838182	1457220
WRLP440	*L. monocytogenes*	1	32	2892224	498279
WRLP443	*L. monocytogenes*	1	32	2892414	498434
WRLP446	*L. monocytogenes*	1	32	2892350	498434
WRLP455	*L. monocytogenes*	1	36	2927486	476657
WRLP459	*L. monocytogenes*	1	39	2927219	475201
WRLP463	*L. monocytogenes*	1	39	2927400	476657
WRLP466	*L. monocytogenes*	1	34	2927379	476657
WRLP468	*L. monocytogenes*	1	36	2927486	476657
WRLP469	*L. monocytogenes*	1	34	2927373	476657
WRLP472	*L. monocytogenes*	1	22	2895033	1495945
WRLP477	*L. monocytogenes*	1	25	2894404	353377
WRLP483	*L. monocytogenes*	1	36	2930302	476657
WRLP486	*L. monocytogenes*	1	37	2927399	476657
WRLP490	*L. monocytogenes*	1	21	2894954	449882
WRLP491	*L. monocytogenes*	1	25	2894404	353377
WRLP494	*L. monocytogenes*	1	37	2927399	476657
WRLP496	*L. monocytogenes*	1	36	2927378	476657
WRLP498	*L. monocytogenes*	1	22	2894543	449882
WRLP499	*L. monocytogenes*	1	36	2927373	476657
WRLP502	*L. monocytogenes*	1	40	2927200	476657
WRLP508	*L. monocytogenes*	1	22	2894549	449882

**Strain ID**	**Species**	**Facility**	**Contigs No.**	**Length (bp)**	**N50**
WRLP512	*L. monocytogenes*	1	36	2927373	476657
WRLP517	*L. monocytogenes*	1	36	2927379	476657
WRLP519	*L. monocytogenes*	1	39	2927364	476657
WRLP520	*L. monocytogenes*	1	39	2927393	476657
WRLP522	*L. monocytogenes*	1	22	2894549	449882
WRLP521	*L. monocytogenes*	1	35	2927534	476657
WRLP525	*L. monocytogenes*	1	22	2894549	449882
WRLP527	*L. monocytogenes*	1	39	2927399	476657
WRLP530	*L. monocytogenes*	1	27	2883618	509939
WRLP533	*L. monocytogenes*	1	31	2883795	509804

Table S2. NCBI Accession numbers used to screen for antimicrobials tolerance determinants and virulence genes.

Gene/ gene region	Accession no.
tetR, tnpABC	HF565366.1
qacH	HG329628
qacC	QXKR01000002.1
emrE	CP001602.2
emrC	LT732640.1
bcrABC	JX023276
cadA1	L28104
cadA2	AADR01000058
LIPI-1, SSI-1, *inlA*	NC_003210
LIPI-3	NZ_JABXLV000000000.1
LIPI-4	FM242711.1

Table S3. GoTerm analysis of select CC155 accessory genes annotated as DNA binding, transcriptional regulator or membrane component.

SeqName	Description	Length	#Hits	e-Value	Similarity mean	#GO	GO IDs	GO Names
group_1835	TetR/AcrR family transcriptional regulator	594	20	3.88E-143	99.75	1	F:GO:0003677	F:DNA binding
group_1029	sigma-70 family RNA polymerase sigma factor	405	20	1.23E-89	99.22	3	P:GO:2000142; F:GO:0003677; F:GO:0016987	P:regulation of DNA-templated transcription, initiation; F:DNA binding; F:sigma factor activity
mtrR	TetR/AcrR family transcriptional regulator	528	20	3.44E-125	97.4	1	F:GO:0003677	F:DNA binding
xerD_2	site-specific integrase	1164	20	0	99.94	3	P:GO:0006310; P:GO:0015074; F:GO:0003677	P:DNA recombination; P:DNA integration; F:DNA binding
group_2252	transposase	282	20	9.79E-59	92.57	4	P:GO:0006313; P:GO:0015074; F:GO:0003677; F:GO:0004803	P:transposition, DNA-mediated; P:DNA integration; F:DNA binding; F:transposase activity
group_2270	recombinase family protein	621	20	6.39E-147	98.82	4	P:GO:0006310; P:GO:0015074; F:GO:0000150; F:GO:0003677	P:DNA recombination; P:DNA integration; F:DNA strand exchange activity; F:DNA binding
mrr	restriction endonuclease	984	20	0	99.97	4	P:GO:0009307; P:GO:0090305; F:GO:0003677; F:GO:0004519	P:DNA restriction-modification system; P:nucleic acid phosphodiester bond hydrolysis; F:DNA binding; F:endonuclease activity
group_1168	XRE family transcriptional regulator	993	20	4.85E-179	98.19	2	F:GO:0003677; C:GO:0016021	F:DNA binding; C:integral component of membrane
group_1510	XRE family transcriptional regulator	264	20	5.39E-55	99.25	1	F:GO:0003677	F:DNA binding
group_306	divergent PAP2 family protein	474	20	1.89E-108	99.97	1	C:GO:0016021	C:integral component of membrane
sauU	MFS transporter	1206	20	0	99.79	4	P:GO:0055085; F:GO:0022857; C:GO:0005886; C:GO:0016021	P:transmembrane transport; F:transmembrane transporter activity; C:plasma membrane; C:integral component of membrane
group_676	hypothetical protein M640_01320	651	20	3.32E-142	99.77	1	C:GO:0016021	C:integral component of membrane

SeqName	Description	Length	#Hits	e-Value	Similarity mean	#GO	GO IDs	GO Names
group_532	competence protein ComG	318	20	9.40E-68	99.33	1	C:GO:0016021	C:integral component of membrane
group_1281	sensor histidine kinase	1740	20	0	99.94	4	P:GO:0000160; P:GO:0016310; F:GO:0000155; C:GO:0016021	P:phosphorelay signal transduction system; P:phosphorylation; F:phosphorelay sensor kinase activity; C:integral component of membrane
group_1466	GAP family protein	258	20	2.84E-50	100	1	C:GO:0016021	C:integral component of membrane

Figure S1. Pairwise SNP matrix.

strain	WRLP477	WRLP472	WRLP525	WRLP491	WRLP508	WRLP522	WRLP490	WRLP498	WRLP410	WRLP370	WRLP367	WRLP417	WRLP416	WRLP434	WRLP360	WRLP446	WRLP443	WRLP440	WRLP422	WRLP459	WRLP512	WRLP468	WRLP466	WRLP394	WRLP469	WRLP520	WRLP519	WRLP521	WRLP517	WRLP463	WRLP455	WRLP486	WRLP494	WRLP499	WRLP527	WRLP483	WRLP496	WRLP502	WRLP377	WRLP378	WRLP382	WRLP380	WRLP354	WRLP386	WRLP530	WRLP533	WRLP408	WRLP409
WRLP477	0	0	0	0	0	1	1	1	2162	2282	2520	2168	2180	2313	2547	9560	1067	1116	1039	1051	1119	1186	1177	1168	1132	1104	1100	1122	1082	1082	1102	1050	1049	1050	1035	1019	1014	9594	9916	1036	1098	1097	1127	1202	1125	1073	1148	9689
WRLP472	0	0	0	0	0	1	1	1	21620	22815	25203	21682	21810	23142	25477	95584	10670	11164	10389	10513	11194	11860	11770	11687	11327	11042	11002	11230	10824	10819	11026	10503	10495	10500	10354	10198	10141	95964	99149	10367	10984	10974	11269	12024	11259	10733	11484	96903
WRLP525	0	0	0	0	0	1	1	1	21630	22825	25213	21684	21810	23142	25476	95552	10668	11162	10388	10511	11192	11856	11767	11684	11325	11038	10997	11225	10822	10818	11024	10497	10492	10498	10353	10197	10140	95937	99140	10363	10982	10972	11265	12021	11255	10731	11480	96881
WRLP491	0	0	0	0	0	1	1	1	21606	22808	25187	21671	21795	23119	25451	95504	10663	11156	10383	10508	11187	11853	11764	11680	11321	11034	10993	11221	10819	10815	11020	10493	10487	10493	10346	10190	10133	95898	99074	10357	10976	10968	11262	12017	11252	10722	11476	96833
WRLP508	0	0	0	0	0	1	1	1	21632	22831	25216	21690	21814	23145	25482	95637	10678	11172	10398	10521	11202	11868	11779	11695	11336	11049	11008	11236	10833	10829	11035	10509	10501	10507	10363	10206	10148	96040	99217	10373	10992	10981	11277	12033	11265	10739	11492	96954
WRLP522	1	1	1	1	1	0	2	2	21614	22814	25193	21681	21801	23132	25463	95576	106676	111611	103885	105106	111938	118585	117688	116857	113269	110398	109988	112270	108243	108181	110242	105005	104917	104987	103539	101963	101394	95919	99129	103630	109814	109733	112678	120236	112551	107285	114820	96859
WRLP490	1	1	1	1	1	2	0	2	21616	22815	25200	21678	21804	23136	25473	95536	10668	11161	10389	10510	11192	11856	11767	11683	11324	11039	10996	11226	10822	10818	11024	10497	10492	10497	10351	10194	10138	95953	99154	10363	10980	10972	11266	12021	11253	10728	11481	96850
WRLP498	1	1	1	1	1	2	2	0	2163	2283	2521	2168	2181	2314	2547	9564	1067	1117	1039	1052	1120	1186	1177	1169	1133	1105	1100	1123	1083	1082	1103	1050	1050	1050	1036	1020	1015	9602	9923	1037	1099	1098	1127	1203	1126	1073	1149	9695
WRLP410	21629	21620	21630	21606	21632	21614	21616	21635	0	313	415	20792	20854	22815	24182	94434	10449	10902	10190	10315	10928	11515	11441	11373	11049	10781	10746	10961	10593	10590	10783	10283	10274	10270	10168	10003	99581	94714	97624	10143	10717	10700	10946	11654	10961	10484	11190	95724
WRLP370	2282	2281	2282	2280	2283	2281	2281	2283	313	0	280	2142	2140	2367	2494	9542	1055	1100	1028	1041	1103	1161	1154	1148	1114	1088	1084	1106	1068	1069	1087	1038	1037	1037	1027	1010	1006	9563	9863	1024	1081	1080	1105	1174	1107	1058	1129	9676
WRLP367	2520	2520	2521	2518	2521	2519	2520	2521	415	280	0	2281	2286	2515	2639	9741	1075	1121	1049	1062	1123	1183	1176	1169	1135	1109	1106	1127	1090	1090	1108	1058	1057	1058	1047	1031	1026	9773	1006	1044	1102	1100	1126	1196	1127	1079	1150	9881
WRLP417	2168	2168	2168	2167	2169	2168	2167	2168	2079	2142	2281	0	245	2124	2241	9426	1044	1091	1018	1030	1093	1154	1145	1139	1106	1079	1075	1097	1060	1060	1078	1029	1028	1030	1017	1003	9961	9466	9746	1013	1071	1069	1097	1167	1098	1049	1119	9567
WRLP416	21807	21810	21810	21795	21814	21801	21804	21810	20854	21401	22863	245	0	21238	22452	94511	10469	10929	10203	10326	10944	11554	11481	11417	11081	10823	10780	10992	10620	10624	10807	10305	10298	10331	10194	10044	99725	94872	97621	10157	10737	10722	10990	11694	11002	10521	11219	95946
WRLP434	2313	2314	2314	2311	2314	2313	2313	2314	2281	2367	2515	2124	2123	0	404	9454	1048	1094	1022	1033	1095	1156	1147	1141	1109	1081	1078	1099	1061	1062	1082	1032	1031	1032	1018	1003	9966	9486	9787	1018	1076	1074	1100	1169	1100	1051	1123	9604

strain	WRLP477	WRLP472	WRLP525	WRLP491	WRLP508	WRLP522	WRLP490	WRLP498	WRLP410	WRLP370	WRLP367	WRLP417	WRLP416	WRLP434	WRLP360	WRLP446	WRLP443	WRLP440	WRLP422	WRLP459	WRLP512	WRLP468	WRLP466	WRLP394	WRLP469	WRLP520	WRLP519	WRLP521	WRLP517	WRLP463	WRLP455	WRLP486	WRLP494	WRLP499	WRLP527	WRLP483	WRLP496	WRLP502	WRLP377	WRLP378	WRLP382	WRLP380	WRLP354	WRLP386	WRLP530	WRLP533	WRLP408	WRLP409

strain	WRLP455	WRLP463	WRLP517	WRLP521	WRLP519	WRLP520	WRLP469	WRLP394	WRLP466	WRLP468	WRLP512	WRLP459	WRLP422	WRLP440	WRLP443	WRLP446	WRLP360
WRLP477	110282	108209	108258	112295	110027	110430	113295	116881	117715	118606	111948	105158	103918	111656	106732	95607	25473
WRLP472	110267	108199	108246	112305	110022	110429	113273	116872	117706	118606	111949	105135	103895	111645	106706	95584	25477
WRLP525	110247	108183	108223	112254	109978	110383	113250	116845	117678	118569	111927	105111	103885	111625	106685	95552	25476
WRLP491	110204	108156	108191	112211	109932	110342	113210	116801	117640	118534	111872	105080	103832	111567	106639	95504	25451
WRLP508	110356	108294	108331	112368	110080	110491	113363	116954	117793	118686	112028	105216	103981	111723	106782	95637	25482
WRLP522	110242	108181	108243	112270	109988	110398	113269	116857	117688	118585	111938	105106	103885	111611	106676	95576	25463
WRLP490	110248	108188	108222	112261	109967	110391	113248	116839	117678	118568	111922	105107	103893	111616	106688	95536	25473
WRLP498	110353	108280	108331	112368	110084	110501	113363	116945	117785	118681	112032	105211	103982	111736	106787	95642	25476
WRLP410	107830	105909	105930	109619	107464	107811	110494	113738	114418	115153	109282	103153	101905	109023	104491	94434	24182
WRLP370	108755	106912	106867	110638	108472	108805	111491	114814	115475	116173	110325	104139	102869	110058	105545	95421	24948
WRLP367	110837	109026	109037	112714	110611	110944	113587	116946	117603	118304	112375	106242	104960	112120	107578	97419	26392
WRLP417	107850	106016	106069	109725	107552	107998	110655	113969	114578	115405	109328	103071	101848	109113	104473	94264	22413
WRLP416	108074	106249	106202	109925	107800	108230	110815	114174	114814	115548	109440	103266	102032	109296	104691	94511	22452
WRLP434	108223	106251	106194	109972	107842	108167	110942	114122	114751	115614	109542	103368	102245	109452	104878	94540	404
WRLP360	109953	108027	107981	111697	109550	109988	112747	115960	116570	117385	111283	105116	104032	111193	106660	96279	0
WRLP446	7861	7645	7719	7827	7709	7870	7927	8233	8228	8295	7744	6778	1887	1814	1794	0	96279
WRLP443	8166	7958	8062	8134	8042	8177	8174	8303	8261	8421	7930	7133	1686	1269	0	1794	106660
WRLP440	7905	7750	7810	7866	7775	7808	7840	7852	7846	7840	7624	6995	1454	0	1269	1814	111193
WRLP422	8254	7864	8030	8137	7981	8145	8251	8343	8367	8390	7894	7088	0	1454	1686	1887	104032
WRLP459	7839	7761	7794	7844	7669	7778	7902	8091	7987	8096	7665	0	7088	6995	7133	6778	105116
WRLP512	952	970	993	903	863	820	777	752	682	682	0	7665	7894	7624	7930	7744	111283
WRLP468	996	965	1113	783	783	816	585	374	353	0	682	8096	8390	7840	8421	8295	117385
WRLP466	968	935	1077	836	800	785	597	368	0	353	682	7987	8367	7846	8261	8228	116570
WRLP394	1003	874	1092	805	821	880	642	0	368	374	752	8091	8343	7852	8303	8233	115960
WRLP469	906	868	1152	889	879	828	0	642	597	585	777	7902	8251	7840	8174	7927	112747
WRLP520	1100	999	1061	970	892	0	828	880	785	816	820	7778	8145	7808	8177	7870	109988
WRLP519	1097	1028	1064	930	0	892	879	821	800	783	863	7669	7981	7775	8042	7709	109550
WRLP521	1006	1019	1005	0	930	970	889	805	836	783	903	7844	8137	7866	8134	7827	111697
WRLP517	1236	1127	0	1005	1064	1061	1152	1092	1077	1113	993	7794	8030	7810	8062	7719	107981
WRLP463	1141	0	1127	1019	1028	999	868	874	935	965	970	7761	7864	7750	7958	7645	108027
WRLP455	0	1141	1236	1006	1097	1100	906	1003	968	996	952	7839	8254	7905	8166	7861	109953
WRLP486	1413	1449	1591	1411	1436	1473	1279	1436	1420	1498	1345	7881	8083	7994	8080	7663	104942
WRLP494	1553	1491	1458	1522	1424	1443	1454	1471	1551	1595	1290	7773	8147	8102	8142	7642	104966
WRLP499	1683	1449	1735	1426	1431	1597	1583	1563	1678	1677	1568	7925	8163	8077	8181	7752	104997
WRLP527	1876	1770	1774	1836	1814	1829	1727	1908	1898	1967	1660	7827	8196	8160	8338	7810	103748
WRLP483	1865	1789	1966	1865	1772	1815	1737	1932	1951	2089	1775	7812	8077	8283	8287	7574	102165
WRLP496	1934	1890	1928	2053	1954	1802	1875	2058	2207	2245	1844	7982	8135	8313	8255	7767	101520
WRLP502	2704	2442	2434	2525	2542	2654	2737	2988	3017	3047	2590	8073	8342	8791	8615	7852	96604
WRLP377	8769	8657	8780	8912	8728	8803	8920	9091	9228	9262	8633	7915	8342	8519	8494	7603	99582
WRLP378	8463	8381	8355	8568	8389	8333	8547	8658	8711	8836	8428	7684	8115	8165	8130	7744	103657
WRLP382	8545	8440	8639	8481	8351	8493	8419	8507	8570	8610	8277	8006	8346	8119	8262	8070	109351
WRLP380	8323	8295	8400	8367	8089	8236	8378	8279	8531	8357	8120	7792	8109	7908	8206	7944	109084
WRLP354	8418	8333	8556	8436	8260	8366	8354	8325	8454	8323	8117	8028	8454	7919	8319	8091	111832
WRLP386	8884	8867	9077	8848	8679	8682	8759	8601	8650	8522	8584	8673	8927	8290	8906	9022	118728
WRLP530	8290	8265	8369	8178	8185	8191	8088	8025	8039	8094	8034	7735	7905	7652	7976	7988	111952
WRLP533	8769	8601	8753	8640	8637	8694	8664	8722	8818	8881	8547	7897	8190	8131	8215	8095	106991
WRLP408	8563	8351	8546	8547	8392	8458	8367	8305	8386	8406	8309	8039	8426	7953	8303	8260	114209
WRLP409	9308	9067	9052	9441	9237	9284	9393	9729	9825	9940	9310	8335	8573	9017	8677	8148	97887

WRLP409	WRLP408	WRLP533	WRLP530	WRLP386	WRLP354	WRLP380	WRLP382	WRLP378	WRLP377	WRLP502	WRLP496	WRLP483	WRLP527	WRLP499	WRLP494	WRLP486
96895	114848	107305	112586	120252	112713	109760	109864	103677	99160	95947	101427	101997	103565	105018	104952	105009
96903	114849	107330	112597	120244	112693	109740	109847	103672	99149	95964	101415	101982	103547	105008	104955	105039
96881	114807	107313	112558	120212	112658	109722	109829	103630	99140	95937	101401	101972	103532	104984	104929	104978
96833	114766	107229	112525	120173	112629	109685	109763	103574	99074	95898	101332	101907	103468	104937	104878	104936
96954	114920	107397	112656	120331	112772	109817	109921	103730	99217	96040	101483	102067	103633	105076	105011	105091
96859	114820	107285	112551	120236	112678	109733	109814	103630	99129	95919	101394	101963	103539	104987	104917	105005
96850	114819	107284	112537	120216	112662	109723	109808	103634	99154	95953	101384	101948	103512	104974	104924	104978
96950	114918	107378	112645	120324	112775	109835	109919	103732	99239	96029	101516	102073	103623	105085	105018	105089
95724	111903	104846	109612	116541	109465	107008	107178	101437	97624	94714	99581	100031	101689	102709	102747	102837
96761	112900	105892	110717	117471	110522	108047	108146	102413	98634	95635	100610	101098	102717	103723	103749	103807
98815	115063	107972	112756	119696	112628	110073	110239	104469	100659	97737	102668	103132	104750	105828	105774	105882
95670	111990	104930	109811	116704	109712	106990	107171	101389	97461	94667	99612	100316	101780	103043	102826	102934
95946	112195	105218	110023	116949	109900	107228	107373	101570	97621	94872	99725	100444	101940	103315	102984	103059
96041	112366	105184	110095	116993	110050	107405	107661	101872	97878	94865	99660	100302	101885	103237	103164	103208
97887	114209	106991	111952	118728	111832	109084	109351	103657	99582	96604	101520	102165	103748	104997	104966	104942
8148	8260	8095	7988	9022	8091	7944	8070	7744	7603	7852	7767	7574	7810	7752	7642	7663
8677	8303	8215	7976	8906	8319	8206	8262	8130	8494	8615	8255	8287	8338	8181	8142	8080
9017	7953	8131	7652	8290	7919	7908	8119	8165	8519	8791	8313	8283	8160	8077	8102	7994
8573	8426	8190	7905	8927	8454	8109	8346	8115	8342	8342	8135	8077	8196	8163	8147	8083
8335	8039	7897	7735	8673	8028	7792	8006	7684	7915	8073	7982	7812	7827	7925	7773	7881
9310	8309	8547	8034	8584	8117	8120	8277	8428	8633	2590	1844	1775	1660	1568	1290	1345
9940	8406	8881	8094	8522	8323	8357	8610	8836	9262	3047	2245	2089	1967	1677	1595	1498
9825	8386	8818	8039	8650	8454	8531	8570	8711	9228	3017	2207	1951	1898	1678	1551	1420
9729	8305	8722	8025	8601	8325	8279	8507	8658	9091	2988	2058	1932	1908	1563	1471	1436
9393	8367	8664	8088	8759	8354	8378	8419	8547	8920	2737	1875	1737	1727	1583	1454	1279
9284	8458	8694	8191	8682	8366	8236	8493	8333	8803	2654	1802	1815	1829	1597	1443	1473
9237	8392	8637	8185	8679	8260	8089	8351	8389	8728	2542	1954	1772	1814	1431	1424	1436
9441	8547	8640	8178	8848	8436	8367	8481	8568	8912	2525	2053	1865	1836	1426	1522	1411
9052	8546	8753	8369	9077	8556	8400	8639	8355	8780	2434	1928	1966	1774	1735	1458	1591
9067	8351	8601	8265	8867	8333	8295	8440	8381	8657	2442	1890	1789	1770	1449	1491	1449
9308	8563	8769	8290	8884	8418	8323	8545	8463	8769	2704	1934	1865	1876	1683	1553	1413
9003	8667	8663	8556	9246	8581	8525	8429	8228	8503	2450	2048	1930	1831	1822	1701	0
8853	8627	8700	8452	9363	8602	8391	8544	8407	8512	2444	1896	1974	1827	1766	0	1701
9050	8806	8792	8565	9303	8654	8532	8575	8460	8619	2639	2102	2035	1885	0	1766	1822
8907	8943	8749	8764	9696	8887	8678	8749	8483	8618	2602	2031	1954	0	1885	1827	1831
8784	8971	8790	8721	9628	8867	8750	8654	8492	8600	2267	2179	0	1954	2035	1974	1930
8805	9002	8900	8791	9866	8954	8841	8871	8515	8629	2396	0	2179	2031	2102	1896	2048
8956	9570	9292	9319	10424	9472	9177	9458	8939	8713	0	2396	2267	2602	2639	2444	2450
8619	8946	8456	8407	2818	2401	2196	2069	2041	0	8713	8629	8600	8618	8619	8512	8503
8688	8621	8416	8313	2153	1812	1800	1582	0	2041	8939	8515	8492	8483	8460	8407	8228
9205	8252	8303	7994	1454	1454	1424	0	1582	2069	9458	8871	8654	8749	8575	8544	8429
9259	8253	8315	7806	1208	947	0	1424	1800	2196	9177	8841	8750	8678	8532	8391	8525
9452	8222	8428	7786	876	0	947	1454	1812	2401	9472	8954	8867	8887	8654	8602	8581
10302	8442	8896	8038	0	876	1208	1454	2153	2818	10424	9866	9628	9696	9303	9363	9246
8983	7652	1069	0	8038	7786	7806	7994	8313	8407	9319	8791	8721	8764	8565	8452	8556
8695	8348	0	1069	8896	8428	8315	8303	8416	8456	9292	8900	8790	8749	8792	8700	8663
2695	0	8348	7652	8442	8222	8253	8252	8621	8946	9570	9002	8971	8943	8806	8627	8667
0	2695	8695	8983	10302	9452	9259	9205	8688	8619	8956	8805	8784	8907	9050	8853	9003
